# Evolutionary Stability of the Shark Snout: Geometric Morphometrics of Ventral Facial Openings

**DOI:** 10.1111/nyas.70323

**Published:** 2026-06-16

**Authors:** Stefano Aicardi, Giacomo Rosa, Alessio Longo, Matteo Bozzo, Beatrice Risso, Elisa Damonte, Andrea Amaroli, Simona Candiani, Sara Ferrando

**Affiliations:** ^1^ DiSTAV Università di Genova Genova Italy; ^2^ NBFC Palermo Italy; ^3^ Department of Comparative Biomedicine and Food Science Università di Padova Padova Italy; ^4^ IRCCS Istituto G. Gaslini Genova Italy

**Keywords:** chondrichthyes, head shape, shark evolution, shark morphology

## Abstract

Chondrichthyans are among the most ancient vertebrates, and their morphology has enabled adaptation to diverse marine environments. In sharks, the head is the functional center for feeding, respiration, and sensory perception, reflecting a balance between phylogenetic constraints and ecological demands. Striking specializations, such as the cephalofoil of hammerhead sharks or the elongated rostrum of sawsharks, illustrate remarkable adaptations. Most studies on head shape have focused on single species or restricted clades, whereas broader analyses have focused on parts of the skull. We analyzed the ventral head morphology of 453 shark species (Selachii), focusing on the relative position of the rostrum, mouth, and nostrils (in a subset of 395 species), using standardized illustrations and landmark‐based geometric morphometrics. Shape variation was related to ecological and anatomical factors. The morphological space reveals a phylogenetic signal, with reconstructed ancestral shapes close to the overall mean configuration, indicating strong morphological conservatism. Evolutionary model fitting supports Brownian motion dynamics with limited divergence from ancestral forms. Correlations with habitat and body size are weak, whereas stronger associations are observed with the number of olfactory lamellae. Our results provide a large‐scale comparative framework of ventral head morphology in sharks, highlighting its evolutionary stability and predominant phylogenetic control.

## Graphical abstract

Landmark‐based geometric morphometrics of 453 shark species reveal that ventral head morphology is conserved across Selachii. Morphospace and ancestral reconstructions support strong phylogenetic control and limited divergence from ancestral conditions. Habitat, swimming mode, and body size show weak effects on shape, whereas olfactory lamellar number is more strongly associated, but this pattern is driven by a few extreme species. Phylogeny is the dominant driver of ventral head morphology in sharks.

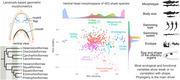

## 1 Introduction

Chondrichthyans represent the earliest diverging extant lineage of jawed vertebrates [1, 2], and their head morphology is deeply tied to their ecology [2, 3, 4, 5, 6, 7, 8, 9, 10, 11]. The head is the site of feeding and respiration but also integrates a wide array of sensory functions and contributes to locomotor efficiency [2, 3, 4, 5, 6, 7, 8, 9, 10, 11]. As such, its form reflects a complex interplay of evolutionary constraints and ecological demands.

Sharks (Selachii) provide striking examples of head specializations. The wide cephalofoil of hammerhead sharks (family Sphyrnidae), for instance, has been proposed to enhance olfactory sampling [6], to accommodate a broader distribution of the ampullae of Lorenzini (electroreceptors), and to improve steering through its hydrodynamic properties [6, 7, 8, 10]. In sawsharks (order Pristiophoriformes), the elongated rostrum is recognized as a sensory structure particularly involved in electroreception and mechanoreception [11]. Variation in the position and shape of the nostrils has also been described as an adaptation to active swimming versus sedentary habits [5].

Some studies addressing shark head morphology have been restricted to single species or to limited taxonomic subsets [5, 6, 7, 8, 9, 10, 11], but more recently wider investigations have addressed different aspects of shark head morphology in an ecological and evolutionary context [12, 13, 14]. These studies adopt a geometric morphometric approach applied to skeletal components of the head, focusing on the neurocranium or the mandible. The morphology of both structures exhibits a strong phylogenetic signal, while also reflecting habitat‐related patterns that are not always straightforward to interpret [13, 14].

In the present study, we take a complementary perspective by shifting the focus from isolated skeletal elements to the external head morphology, considered exclusively in the ventral view. Yet, the taxonomic breadth and global distribution of sharks across a wide range of marine environments make comprehensive sampling of physical specimens logistically demanding. One way to overcome this limitation is the use of pictographic datasets. Standardized illustrations from authoritative ichthyological references have already proven reliable for geometric morphometrics at large taxonomic scales. Thomson and Simanek [15] analyzed body shapes of sharks using the pictographs provided by Bigelow and Schroeder [16]. More recently, Siders et al. [17] provided the first empirical validation of pictographs use by assessing the reliability of these images for large‐scale morphological analyses, using outline‐based geometric morphometrics on shark pictographs from multiple scientific sources. Sternes and Shimada [18] and Sternes et al. [19] applied landmark‐based geometric morphometrics to pictographs from *Sharks of the World: A Fully Illustrated Guide* [20]. In addition, the geometric morphometric analysis proposed by Sternes and Shimada [18] was later applied in a subsequent study by Gayford et al. [21], which consequently conducted its analyses using the same set of illustrations. Here, we extend this pictographic approach to a previously overlooked anatomical target: the ventral view of the shark head, with particular attention to the snout, mouth, and, when ventrally positioned, nostrils. This two‐dimensional perspective necessarily excludes many aspects of head morphology, which have been addressed elsewhere [13, 14], or remain to be explored, but it captures features that are functionally relevant and consistently represented across taxa. By assembling a dataset that spans nearly all extant Selachii, we apply geometric morphometrics to examine how these traits vary across phylogeny, correlate with ecological descriptors such as habitat and swimming mode, and relate to other aspects of head anatomy, such as sensory organ size and shape.

Although detailed specimen‐based analyses remain essential for resolving species‐level variation, our results provide a broad evolutionary overview of selachian head diversification, adding to the understanding of how phylogeny and ecology have shaped the diversity of shark head morphology.

## 2 Materials and Methods

### 2.1 Landmarks and Semilandmarks Datasets

To gather a comprehensive dataset regarding the shape of the snout, mouth, and nostrils of Selachii, landmarks were positioned on anatomically accurate drawings from the book *Sharks of the World: A Fully Illustrated Guide* [20]. Illustrations from this book have previously been used to analyze the body shape of sharks through a geometric morphometric approach [18, 19, 21]. The images of the ventral view of the heads of Selachii from the book were digitized and then landmarks and homologous curves were collected using ImageJ [22].

**FIGURE 1:**
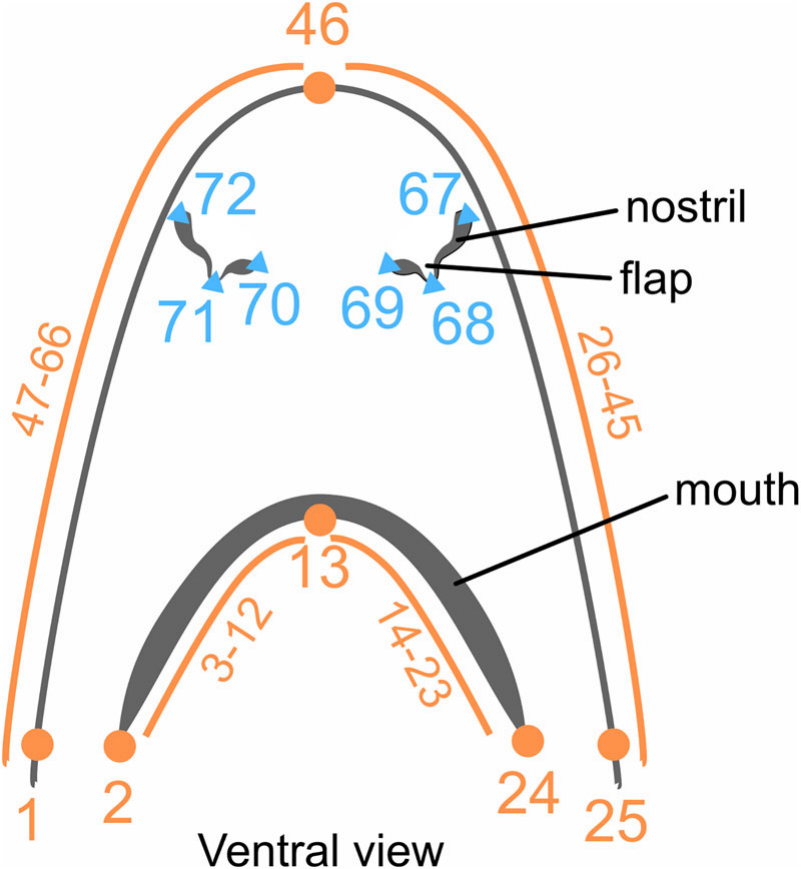
General drawing of the head of a shark, ventral view. Orange circles indicate the six landmarks included in both the RO and RNO datasets; three are located on the snout edge and three along the mouth. Orange lines indicate the four homologous curves along which the 60 semilandmarks—also present in both datasets—were selected; these curves run along the snout and the oral fissure. Blue triangles indicate the six landmarks included only in the RNO dataset; they correspond to the medial and lateral points of the nostrils and the distal tip of the nasal flaps.

Two different datasets were obtained which differ in the presence or absence of six landmarks that identify the medial and lateral points of the nostrils and the distal tip of the flap that divides the inlet and outlet canals of the nostrils. As the nostrils are not ventrally visible in all the species, the number of species in the two datasets is different. The rostro–oral dataset (RO dataset) comprises 6 landmarks and 4 homologous curves on which 60 semilandmarks were positioned for 453 species out of 607 valid species of Selachii (Eschmeyer's Catalog of Fishes, Online Version^1^, Updated August 15, 2025). The rostro–naso–oral dataset (RNO dataset) comprises 12 landmarks and 4 homologous curves where 60 semilandmarks were positioned for 395 species (Figure 1 and Table 1). The landmarks and semilandmarks of the RO dataset are a subset of those of the RNO dataset, whereas the species present in the RNO dataset are a subset of those of the RO dataset. To obtain a cleaner view of the shape of the snout of Selachii, further subsets were obtained to perform some of the analyses by deleting species with a very divergent shape of the snout, namely, the order Pristiophoriformes (sawsharks, seven species in the complete dataset) and the family Sphyrnidae (hammerhead sharks, eight species in the complete dataset). The included species are reported in Table S1, whereas a summary of the datasets and their distribution among orders is reported in Table 1.

**TABLE 1:** Datasets used in this study and their taxonomic coverage.

Dataset	Number of species	Description	Cohort and superorders	Orders
RO dataset	453	All the available species (6 landmarks + 60 semilandmarks related to snout and mouth shape)	Selachii 453/607 spp. 75%Galeomorphii 317/422 spp. 75%Squalomorphii 136/185 spp. 74%	Carcharhiniformes 73% Echinorhiniformes 100% Heterodontiformes 90% Hexanchiformes 86% Lamniformes 94% Orectolobiformes 80% Pristiophoriformes 70% Squaliformes 77% Squatiniformes 46%
RNO dataset	395	All the available species with ventral nostrils (6 landmarks + 60 semilandmarks related to snout and mouth shape + 6 landmarks related to nostrils)	Selachii 395/607 spp. 65%Galeomorphii 272/422 spp. 64%Squalomorphii 123/185 spp. 66%	Carcharhiniformes 73% Echinorhiniformes 100% Heterodontiformes 0% Hexanchiformes 57% Lamniformes 88% Orectolobiformes 2% Pristiophoriformes 70% Squaliformes 77% Squatiniformes 0%
RO‐sp dataset	438	RO dataset without Sphyrnidae and Pristiophoriformes (6 landmarks + 60 semilandmarks related to snout and mouth shape)	Selachii 438/607 spp. 72%Galeomorphii 309/422 spp. 73%Squalomorphii 129/185 spp. 70%	Carcharhiniformes 71% Echinorhiniformes 100% Heterodontiformes 90% Hexanchiformes 86% Lamniformes 94% Orectolobiformes 80% Pristiophoriformes 0% Squaliformes 77% Squatiniformes 46%
RNO‐sp dataset	380	RNO dataset without Sphyrnidae and Pristiophoriformes (6 landmarks + 60 semilandmarks related to snout and mouth shape + 6 landmarks related to nostrils)	Selachii 380/607 spp. 63%Galeomorphii 264/422 spp. 63%Squalomorphii 116/185 spp. 63%	Carcharhiniformes 71% Echinorhiniformes 100% Heterodontiformes 0% Hexanchiformes 57% Lamniformes 88% Orectolobiformes 2% Pristiophoriformes 0% Squaliformes 77% Squatiniformes 0%

Abbreviations: RNO dataset, rostro–naso–oral dataset; RO dataset, rostro–oral dataset.

### 2.2 Geometric Morphometrics

Procrustes superimposition and the principal component analysis (PCA) were used to obtain shape variation in the two datasets (RO and RNO) of Selachii to describe the morphospace of their RNO region. The package *geomorph* (v4.0.1) [23] was used in the R environment (R v4.1.2‐2021‐11‐01—“Bird Hippie”). Semilandmarks on curves were allowed to slide during generalized Procrustes analysis to minimize thin‐plate spline (TPS) bending energy [24, 25], following the standard implementation in geomorph (gpagen).

A maximum clade credibility (MCC) tree of the selected species was generated in TreeAnnotator using the set of 100 trees downloaded from VerLife (https://vertlife.org/). These 100 trees are representative of the full set of 1000 fully resolved trees, including 10 fossil taxa (Figure S1 and File S1). For the geometric morphometrics of the RO dataset, which has the highest taxonomic coverage, the phylogenetic signal was evaluated with the function *physignal()*. Furthermore, to assess the evolutionary models underlying shape variation, we fit three alternative models of trait evolution—Brownian motion (BM), Ornstein–Uhlenbeck (OU), and early burst (EB)—to the first two PCs of the Procrustes‐aligned coordinates using the package *mvMORPH* (v1.2.1 in the R environment 4.5.1 (2025‐06‐13 ucrt)—“Great Square Root”). PC1 and PC2 together explained ∼87% of total shape variance, whereas PC3 accounted for ∼7%. Preliminary tests showed that including PC3 resulted in unstable likelihood optimization, poorly identified parameters, and inconsistent estimates in OU models, behavior typical of low‐variance and noise‐dominated dimensions in geometric morphometrics. Therefore, only the major axes of variation were retained to ensure reliable phylogenetic modeling. The sample‐size corrected Akaike information criterion (AICc) and the ΔAICc values were used to evaluate the relative fit of the models, with lower AICc scores indicating better support.

Ancestral shapes for selected clades were estimated from the RO dataset. Ancestral landmark coordinates at key nodes (Selachii, Galeomorphii, and Squalomorphii) were reconstructed using maximum likelihood under a BM model along branches of the MCC tree, implemented with the *fastAnc()* function in phytools (ver. 2.0) [26]. In this model, landmark coordinates change along branches according to a multivariate normal distribution, with variance proportional to branch length; no additional model parameters were specified. Following the reconstruction of ancestral states, the shapes of all three nodes were compared to the overall mean shape using TPS visualizations generated with *plotRefToTarget()*. These reconstructions are point estimates only and should be interpreted as visual proxies for ancestral morphology. Due to the absence of fossil taxa, uncertainty increases toward the root, and the results do not capture potential variability or rate heterogeneity among lineages. In the RNO dataset, the covariation between different modules was assessed. Two alternative sets of modules were first defined and tested with *modularity.test()*. The first set divided the landmarks and semilandmarks into 2 modules: a lateral 1 (snout and lateral point of the nostrils, 5 landmarks and 40 semilandmarks) and a medial 1 (mouth, medial point of the nostrils, and flap of the nostril, 7 landmarks and 20 semilandmarks). The second set divided the landmarks into 3 modules: snout (3 landmarks and 40 semilandmarks), nostrils (6 landmarks), and mouth (3 landmarks and 20 semilandmarks). These alternative configurations were designed to reflect both anatomical boundaries and plausible functional subdivisions, rather than exhaustively test all possible landmark combinations. The same module configurations were tested using two‐block partial least‐squares (r‐PLS) analysis within a geometric morphometric framework, including phylogenetic information, to evaluate covariation between modules while accounting for shared ancestry. The configurations tested in the r‐PLS analyses were snout and mouth versus nostrils, snout versus nostrils and mouth, snout and nostrils versus mouth, and lateral versus medial modules (Figure S2). The covariation between modules, both with and without phylogeny, was compared between Galeomorphii and Squalomorphii using the function *compare.pls()*.

To test for convergence among taxa, the R package convevol (ver. 2.2.1) was used. In particular, *Ct* measures [27] were calculated and their significance was assessed using 1000 simulations under a BM model of evolution, focusing on convergence between the family Orectolobidae and the order Squatiniformes. To reduce the number of variables included in the convergence test, a PCA based only on the six fixed landmarks, excluding the 60 semilandmarks, was performed (Figure S3). The exclusion of semilandmarks slightly altered the morphospace configuration, particularly for Sphyrnidae. However, the region of the morphospace, where Orectolobidae and Squatiniformes overlap, was preserved after removing the semilandmarks. Therefore, *Ct* measures were calculated using the Procrustes alignment of the six fixed landmarks only.

### 2.3 Relative Swimming Speed, Swimming Type, and Habitat

Swimming performance of fish is influenced by body form [10, 15]; thus, the head morphology could be correlated to the swimming speed [8] and/or to the swimming type [15]. The relative swimming speed (R‐speed) of shark species in the datasets was calculated using the method developed by Sambilay [28]. This method allows calculation of the R‐speed of a fish (both sharks and teleosts) expressed as body length/second (*L*/s) using the following model:

$$\mathrm{Log}\;\left(\frac{L}{s}\right)=0.616-0.3804\mathrm{Log}\left(L\right)+0.3478\left(\frac{h^{2}}{s}\right)+0.7621\left(M\right)$$

where *L* is the standard length in cm of the fish, *h* is the height of the caudal fin, and *s* is the surface area of the caudal fin; *M* is the swimming mode, and it is 0 for sustained and 1 for burst speeds. Standard length was estimated by combining total length values retrieved from FishBase with species‐specific TL‐to‐SL proportions derived from the illustrations in *Sharks of the World: A Fully Illustrated Guide* [20].

The *h* ^2^/*s* term was called aspect ratio by Sambilay [26] and caudal fin aspect ratio or CFAR by Iliou et al. [29]. The latter paper recently validated the study of Sambilay for sharks, by using measured swimming speed of different species and stating that the swimming speed is positively related to CFAR. For the 453 species of sharks of the present study, the R‐speed was calculated. The *L* of all the species was obtained from FishBase [30], whereas the CFAR was calculated by measuring the drawings on *Sharks of the World: A Fully Illustrated Guide* [20].

Sternes and Shimada [18] found that the body forms almost perfectly correlated with different swimming types (swim types), as indicated by Maia et al. [31] and assigned 359 species out of the 453 here analyzed into anguilliform, carangiform and thunniform swimming types.

Moreover, habitat can be correlated to morphology of shark [18, 32]; therefore, 416 species were assigned to three groups (ecotypes): benthic, benthopelagic, and pelagic, as previously done by Sternes et al. [32] and Gayford et al. [21]. The calculated R‐speeds, the swim types [18], and the habitat [21, 32] are available in Table S1.

### 2.4 Size and Shape of the Body

To explore possible allometric influences on snout shape, maximum total length (maxTL) was used as a proxy for species size. The maxTL was retrieved for all 453 species from FishBase [30] with few exceptions. The maxTL of a few species was not available in FishBase; those values were taken from Sternes and Shimada [18] or from Shark Reference [33].

The shape of the ventral snout and mouth contributes to the anterior body contour and hydrodynamic profile [10], making it reasonable to test for associations between RO morphology and overall body form. The subdivision in three categories of different body shapes (morphotype) was obtained from Gayford et al. [21], and it was available for 417 species out of the 453 here analyzed. Categories A1, A2, and B were obtained by Gayford et al. [21], as a refinement, in a phylogenetic context, of the subdivision in two morphotypes (Groups A and B) in Sternes and Shimada [18], based on the geometric morphometrics applied on the side view of the whole body of sharks. The general body shape was the main source of difference between Group A (elongated‐fusiform as well as dorsoventrally flattened sharks) and Group B (stout deep‐bodied fusiform sharks), although these were related to different positions of fins [18]. The categories A1 and A2 are distinguished by the relative aspect ratio of the caudal fin [21]. The maxTL [18, 30, 33] and the morphotypes [18, 21] are available in Table S1.

### 2.5 Sensory Organs

The head houses major sensory organs, which may occupy a substantial proportion of its volume and might also affect its shape. Some datasets were already available in the literature, and they were used to test this hypothesis, at least for the ventral profile and structures considered in this study. Relative eye size (R‐eyeSize) was obtained from Lisney and Collin [34]. For each of the 31 species shared between their dataset and the present study, eye volume was estimated by approximating the eye as a sphere on the basis of its diameter. This volume (cm^3^) was then divided by body mass (kg) to calculate R‐eyeSize. *Galeus boardmani*, from Lisney and Collin [34], was updated to its valid name, *Figaro boardmani*, according to Eschmeyer's Catalog of Fishes.

Data on the size of the olfactory organ in sharks are fragmentary in the literature. However, another morphological trait of the shark olfactory organ, the lamellar number (N‐lam), is available for 53 species included in the dataset of the present study [35, 36]. The shark olfactory organ is consistently lamellar in structure, with each lamella representing a fold of olfactory mucosa extending from the central support of the organ to the connective capsule that envelops it.

The N‐lam is relatively constant (±10%–12%) among adult specimens of the same species [35] and is defined as the average number of lamellae in a single olfactory organ. The general shape of the head and, in particular, the general shape and relative position of the nostrils interplay with the olfactory organ defining the water current on the sensory surface [37, 38, 39]. The variables R‐eyeSize [34] and N‐lam [35, 36], available for each species retrieved from the literature, are collected in Table S1.

### 2.6 Correlations

Associations between shape variation and variables were tested using phylogenetic generalized least‐squares models in a Procrustes framework, as implemented in the function *procD.pgls()* in the R package *geomorph*. For each variable, analyses were conducted on the subset of species for which the variable was available, and geometric morphometric analyses were repeated accordingly.

Shape data were used as multivariate response variables; continuous explanatory variables (R‐speed, Log maxTL, Log R‐eye Size, and Log N‐lam) and categorical explanatory variable (swim type, morphotype, and ecotype) were each tested in a separate model. Phylogenetic nonindependence was accounted for by incorporating a BM‐based phylogenetic covariance structure derived from the species phylogeny. Statistical significance was assessed using residual randomization permutation procedures (RRPP) with 999 permutations.

## 3 Results

### 3.1 Geometric Morphometrics of the RO Region in 453 Species of Selachii

#### 3.1.1 Shape Variation

**FIGURE 2:**
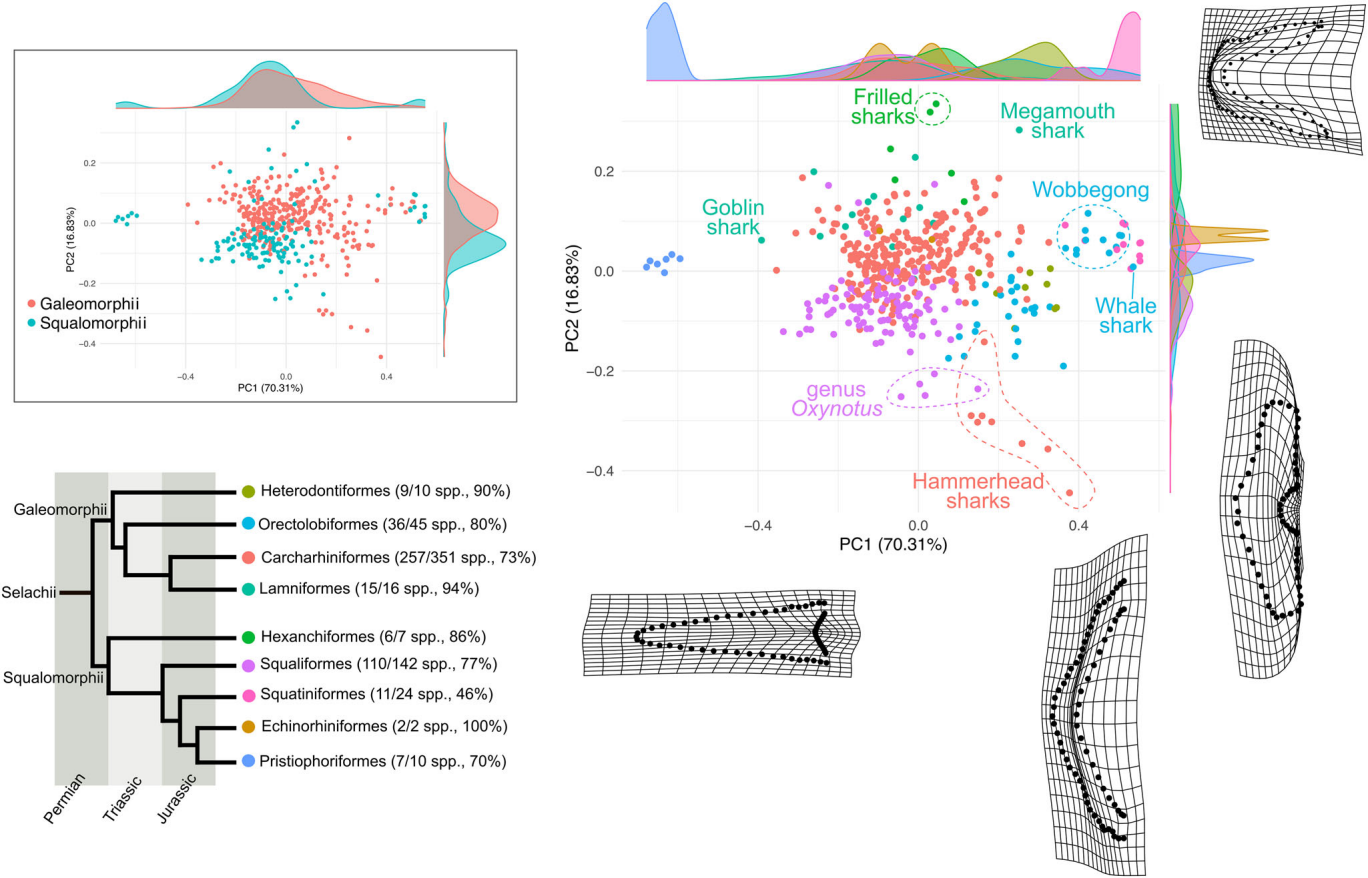
Geometric morphometrics of the rostro–oral region of Selachii (RO dataset, 453 species). Top‐left box: scatterplots with the two superorders Galeomorphii and Squalomorphi highlighted. Main figure: same scatterplots with orders highlighted. Thin‐plate spline deformation grids represent transformations of species with low and high values for PC1 (*Pliotrema warreni* and *Squatina nebulosa*, respectively) and PC2 (*Eusphyra blochii* and *Chlamydoselachus anguineus*, respectively). Some taxa in the scatterplot are labeled Goblin shark (*Mitsukurina owstoni* Jordan, 1898), the genus *Oxynotus*, the families Orectolobidae (Wobbegongs, 11 species of the genus *Orectolobus*, *Sutorectus tentaculatus* (Peters, 1864), and *Eucrossorhinus dasypogon* (Bleeker, 1867)), and Sphyrnidae (hammerhead sharks, seven species of the genus *Sphyrna* and *Eusphyra blochii* (Cuvier, 1816)), the frilled sharks (the only two species of the genus *Chlamydoselachus)*, the megamouth shark (*Megachasma pelagios* Taylor, Compagno and Struhsaker, 1983), and the whale shark (*Rhincodon typus* Smith, 1828). The color legend of orders also contains the phylogenetic tree of Selachii: Superorders Galeomorphii and Squalomorphii and all the orders are indicated. The time of branching is just indicative and not meant to be rigorous (modified from Naylor, 2004 and Torralba Sáez et al. 2024). The number of valid species for each order was obtained from the Eschmeyer's Catalog of Fishes (Online Version, Updated August 15, 2025). The number of species and the percentage of the total number are reported for each order.

The first two principal components (PC1 and PC2) of the PCA, describing the morphospace of the RO region in 453 species of Selachii, explain 70.3% and 16.8% of the total variation, respectively (Figure 2). Selachii are densely distributed in the central region of the morphospace and are fairly clearly separated into two groups corresponding to Galeomorphii and Squalomorphii. This separation is mainly visible along the PC2 axis, as shown by the kernel density distribution, despite PC2 explaining a relatively small proportion of the variance. Outside the central region, a few small groups of species occupy the extremes of the morphospace. Most Squalomorphii are concentrated in the central region but along PC1 they show greater dispersion than Galeomorphii, spanning from the minimum (order Pristiophoriformes) to the maximum (order Squatiniformes). Along PC2, Squalomorphii reach the maximum with the genus *Chlamydoselache*, while extending only slightly toward the minimum represented by the genus *Oxynotus*. Galeomorphii are more tightly distributed, with the order Orectolobiformes and the family Sphyrnidae extending toward the PC1 maximum and PC2 minimum, respectively (Figure 2). The most speciose orders of the Galeomorphii and Squalomorphii, Carcharhiniformes and Squaliformes, respectively, are densely represented in the central region of the morphospace.

Within Galeomorphii, the order Orectolobiformes appears to be divided into two distinct regions. One of these, including the family Orectolobidae (wobbegongs) and the whale shark *Rhincodon typus* Smith, 1828, partially overlaps with the order Squatiniformes (Squalomorphii).

The other Orectolobiformes, comprising all other families, partially overlaps with Heterodontiformes (Galeomorphii). Orectolobiformes and Heterodontiformes, the basalmost branches of Galeomophii, are well separated from Carcharhiniformes and even more from Lamniformes. Within Squalomorphii, the orders occupy separated zones of the morphospace, with Squatiniformes and Pristiophoriformes at the two ends of PC1 and have similar values of PC2 (Figure 2). The shape changes illustrated by the landmarks and semilandmarks along the axes (Figure S4) are highly affected by the presence of the hammerhead sharks (family Sphyrnidae) and of the sawsharks (order Pristiophoriformes). The PCA for the dataset without these 15 species (RO‐sp dataset, devoid of 8 hammerhead sharks and 7 sawsharks) leads to an even higher proportion of variation explained by PC1 (71.97% of variation) and PC2 (18.23%) (Figure S5) and more easily interpretable maximum and minimum shapes along the axes (Figure S4). PC1 is mainly influenced by snout width, reflecting whether the snout is more slender or broader, and by the consequent shape of the mouth. In contrast, PC2 is primarily influenced by the relative position of the mouth to the snout tip, that is, whether it is placed more anteriorly or posteriorly. The observed phylogenetic signal from Procrustes shape variables for the RO dataset (453 species) was *K* = 0.7621, *p* value = 0.001. To evaluate evolutionary dynamics of shape, we compared the fit of BM, OU, and EB models across the first two principal components. Model comparison based on AICc indicated that a single‐rate BM model (BM1) provided the best fit to the data, whereas EB and OU1 models showed ΔAICc values slightly above 2. Overall, results support a BM‐like evolutionary process (Table 2).

**TABLE 2:** Comparison of evolutionary models fitted to the first two principal components using corrected Akaike information criterion (AICc).

Model	logLik	*k* parameters	AICc	ΔAICc	Akaike weight
BM1	1032.212	5	−2054.357	0.000	0.584
OU1	1034.204	8	−2052.248	2.109	0.204
EB	1032.212	6	−2052.330	2.027	0.212

*Note*: The models tested were single‐rate Brownian motion (BM1), Ornstein–Uhlenbeck (OU1), and early burst (EB).

**FIGURE 3:**
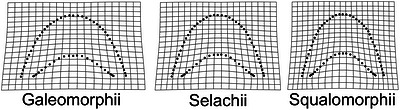
Thin‐plate spline deformation grids showing the reconstructed ancestral shapes for the main selachian clades. Shapes represent the estimated ancestral configurations of the RO dataset (453 species) for Galeomorphii, Selachii (root), and Squalomorphii. Each is visualized as deformation from the overall mean shape of the respective dataset.

Convergence between the family Orectolobidae (11 species in the dataset) and the order Squatiniformes (11 species in the dataset) was assessed using *Ct* measures under 1000 simulations of BM. The overall *Ct*1 value was 0.608 (*p* < 0.001), indicating a reduction of phenotypic distance between the two clades relative to their maximum historical divergence. *Ct*2, *Ct*3, and *Ct*4 values were 0.148 (*p* = 0.002), 0.133 (*p* = 0.010), and 0.004 (*p* = 0.003), respectively. All *Ct* measures were significantly greater than expected under BM, supporting a significant pattern of morphological convergence between the two groups. Ancestral shape reconstructions for Selachii, Squalomorphii, and Galeomorphii are in Figure 3. All three shapes closely resemble the overall mean configuration, with Galeomorphii appearing slightly broader and Squalomorphii slightly narrower, whereas Selachii is nearly identical to the mean.

#### 3.1.2 Phylogenetic Generalized Linear Model (PGLM)

The results of the PGLM analysis for all the variables are reported in Table 3. The log‐transformed maxTL of the species and the ecotype shows a significant correlation with the shape of snout and mouth (PCA of the RO dataset with 453 species for the maxTL and with 416 species for the ecotype), although this correlation accounts for only 1.7% and 2.5% of the variance, respectively (Table 3). Moreover, the log‐transformed N‐lam (53 species) shows a significant correlation with snout and mouth shape, explaining 21% of the variance. To further assess the correlation between RO shape and N‐lam, we tested the PCA of the RO‐sp dataset. The Pristiophoriformes were already absent in the RO dataset for this variable, as their N‐lam is not available. The exclusion of the eight species belonging to the family Sphyrnidae allows us to verify whether the morphological features of this family drive the significance of the correlations. Hammerhead sharks (Sphyrnidae) have a distinctive head shape—strongly expanded laterally—and an extremely high number of lamellae in the olfactory organ [6]. When Sphyrnidae are excluded, the correlation is reduced to 8%, but the *p* value indicates it is no longer significant (Table 3).

**TABLE 3:** Summary of Phylogenetic Generalized Linear Model (PGLM) results for Procruste shapes in relation to different explanatory variables.

	RO dataset		RNO	
Shape ∼ Log maxTL	***R* ^2^ 0.017** ***F* 7.8638** ***Z* 2.9976** ***p* value 0.002** **453 sp**.		***R* ^2^ 0.032** ***F* 12.98** ***Z* 4** ***p* value 0.001** **395 sp**.	
Shape ∼ R‐speed	*R* ^2^ 0.004 *F* 1.5954 *Z* 0.99574 *p* value 0.17 453 sp.		*R* ^2^ 0.003 *F* 1.1495 *Z* 0.56114 *p* value 0.312 395 sp.	
Shape ∼ Log R‐eyeSize	*R* ^2^ 0.021 *F* 0.6085 *Z* −0.14125 *p* value 0.554 31 sp.		*R* ^2^ 0.014 *F* 0.3531 *Z* −0.99195 *p* value 0.834 28 sp.	
Shape ∼ Log N‐lam	***R* ^2^ 0.21** ***F* 13.727** ***Z* 3.3583** ***p* value 0.001** **RO 53 sp**.	*R* ^2^ 0.080 *F* 3.7499 *Z* 1.4173 *p* value 0.079 RO‐sp 45 sp.	***R* ^2^ 0.18** ***F* 11.429** ***Z* 3.4808** ***p* value 0.001** **RNO 53 sp**.	*R* ^2^ 0.079 *F* 3.7063 *Z* 1.516 *p* value 0.066 RNO‐sp 45 sp.
Shape ∼ swim type	*R* ^2^ 0.003 *F* 0.4983 *Z* −0.6976 *p* value 0.761 359 sp.		*R* ^2^ 0.003 *F* 0.4565 *Z* −1.0131 *p* value 0.844 323 sp.	
Shape ∼ morphotype	*R* ^2^ 0.002 *F* 0.5006 *Z* −0.92163 *p* value 0.821 417 sp.		*R* ^2^ 0.005 *F* 0.8549 *Z* −0.054159 *p* value 0.515 373 sp.	
Shape ∼ ecotype	***R* ^2^ 0.025** ***F* 5.3275** ***Z* 2.9719** ***p* value 0.002** **416 sp**.		***R* ^2^ 0.036** ***F* 6.9474** ***Z* 3.503** ***p* value 0.001** **372 sp**.	

Abbreviations: MaxTL, maximum total length; N‐lam, number of lamellae in the olfactory organ; R‐eyeSize, relative size of the eye (cm^3^/kg); R‐speed, relative swimming speed (body length/second).

*Note*: Values associated with statistically significant *p*‐values are shown in bold.

### 3.2 Geometric Morphometrics of the RNO Region in 395 Species of Selachii

#### 3.2.1 Shape Variation

The first two principal components (PC1 and PC2) of the PCA, describing the morphospace of the RNO region in 395 species of Selachii, explain 43.4% and 27.1% of the total variation, respectively (Figure S6a,b). Galeomorphii and Squalomorphii largely overlap, although they tend to spread almost symmetrically toward different regions of the morphospace. PC1 is mainly influenced by overall head shape, particularly by relative rostrum length and head width. Along this axis, the mouth position shifts markedly, moving rostrally from low to high PC1 values. As the nostrils move posteriorly with rostrum shortening and tend to spread as the head becomes wider, the mouth–nostril distance decreases toward higher PC1 values. In contrast, head width has little influence on PC2, whereas rostrum shortening plays a major role, together with an anterior shift of the nostrils and, to a much lesser extent, of the mouth. Mouth shape changes slightly along PC1, from a more linear to a more curved outline, whereas it remains almost constant along PC2 (Figure S4).

The PCA for the dataset without the eight hammerhead sharks and the seven sawsharks explains 51.45% and 21.5% of the variation by PC1 and PC2, respectively (Figure S6c,d). Changes along PC1 are similar to those observed in the complete dataset. In contrast, PC2 differs markedly: Increasing PC2 values are associated with a posterior shift of both the nostrils and the mouth, together with a pronounced change in rostrum shape, which shifts from a flat to a pointed profile (Figure S4).

#### 3.2.2 Phylogenetic Generalized Linear Model

The results of the PGLM analysis for all the variables are reported in Table 3. The log‐transformed maxTL of the species and ecotype again shows a significant correlation with the shape of snout, nostrils, and mouth (PCA of the RNO dataset with 395 species for the maxTL and with 372 species for the ecotype). The correlation accounts again for a small amount of variance, but in both cases, it is higher than when not considering the nostril shapes: 3.2% for the maxTL and 3.6% for the ecotype. Again, the log‐transformed N‐lam (53 species) shows a significant correlation with the shape of snout, nostrils, and mouth, explaining 18% of the variance. As stated before, the exclusion of the eight species belonging to the family Sphyrnidae allows us to verify whether the morphological features of this family drive the significance of the correlations. When the hammerhead sharks are excluded, the correlation decreases, and it is not statistically significant (Table 3).

#### 3.2.3 Covariation of Modules

The covariance ratio test indicates that the three‐module configurations (CR = 0.59, *p* value = 0.001, effect size = −5.56) shows lower between‐module covariance than the two‐module configurations (CR = 0.75, *p* value = 0.001, Effect size = −8.89), thus providing stronger support for a three‐module organizations. Although the separation between the two modules (lateral vs. medial) is less pronounced, this configuration is still statistically significant. Therefore, both modular hypotheses were retained for the subsequent analysis. The r‐PLS analyses for all modules (Figure S2), whether considering phylogeny or not, are reported in Table 4. Overall, the analyses revealed significant covariation among snout, nostril, and mouth shape. Covariation values are consistently lower when phylogeny is taken into account, indicating that part of the observed covariation is explained by shared evolutionary history. Nevertheless, *p* values always indicate significant correlations, and phylo‐r‐PLS values remain relatively high even after phylogenetic correction. In general, nostrils show stronger correlations with overall shape than the mouth. Squalomorphii exhibit higher r‐PLS values only when phylogeny is not considered, suggesting a stronger phylogenetic effect in this clade compared to Galeomorphii (Table 4). The covariation between modules was compared between Galeomorphii and Squalomorphii, and the differences reported in Table 4 are not statistically significant without considering the phylogeny, whereas three of them become significantly different between the two superorders after considering phylogeny (Table 4).

**TABLE 4:** Two‐block partial least‐squares analyses of Procrustes shape variables and covariation comparison between the two superorders.

	Selachii	Galeomorphii	Squalomorphii	Covariation comparison between Galeomorphii and Squalomorphii
Snout + mouth vs. nostrils—12	r‐PLS 0.89 Effect size (*Z*) 9.6733	r‐PLS 0.872 Effect size (*Z*) 9.0017	r‐PLS 0.919 Effect size (*Z*) 6.2205	Effect sizes for pairwise differences in PLS effect size: 0.83 *p* value 0.40
Snout + mouth vs. nostrils (phylogenetically corrected)—12	r‐PLS 0.842 Effect size (*Z*) 9.0246	r‐PLS 0.835 Effect size (*Z*) 7.7528	r‐PLS 0.887 Effect size (*Z*) 6.5941	Effect sizes for pairwise differences in PLS effect size: 0.51 *p* value 0.61
Snout vs. nostrils + mouth—56	r‐PLS 0.825 Effect size (*Z*) 10.617	r‐PLS 0.847 Effect size (*Z*) 8.8165	r‐PLS 0.917 Effect size (*Z*) 7.1572	Effect sizes for pairwise differences: 0.42 *p* value 0.67
Snout vs. nostrils + mouth (phylogenetically corrected)—56	r‐PLS 0.789 Effect size (*Z*) 8.9947	r‐PLS 0.81 Effect size (*Z*) 7.1332	r‐PLS 0.736 Effect size (*Z*) 6.6622	Effect sizes for pairwise differences: 2.44 *p* value 0.015*
Snout + nostrils vs. mouth—34	r‐PLS 0.756 Effect size (*Z*) 9.4094	r‐PLS 0.8 Effect size (*Z*) 8.5586	r‐PLS 0.787 Effect size (*Z*) 5.8207	Effect sizes for pairwise differences in PLS effect size: 0.28 *p* value 0.77
Snout + nostrils vs. mouth (phylogenetically corrected)—34	r‐PLS 0.721 Effect size (*Z*) 7.719	r‐PLS 0.732 Effect size (*Z*) 7.3573	r‐PLS 0.658 Effect size (*Z*) 5.5646	Effect sizes for pairwise differences in PLS effect size: 2.49 *p* value 0.013*
Snout + lateral point of the nostrils vs. mouth + medial point of the nostrils + flaps—78	r‐PLS 0.784 Effect size (*Z*) 10.8687	r‐PLS 0.816 Effect size (*Z*) 9.1243	r‐PLS 0.883 Effect size (*Z*) 6.7771	Effect sizes for pairwise differences in PLS effect size: 0.17 *p* value 0.87
Snout + lateral point of the nostrils vs. mouth + medial point of the nostrils + flaps (phylogenetically corrected)—78	r‐PLS 0.751 Effect size (*Z*) 8.1107	r‐PLS 0.765 Effect size (*Z*) 6.3199	r‐PLS 0.68 Effect size (*Z*) 6.2745	Effect sizes for pairwise differences in PLS effect size: 3.15 *p* value 0.002**

*Note*: Analyses were performed on the RNO dataset (395 species), with module definitions shown in Figure S2. The two‐block partial least‐squares analyses were based on 1000 random permutations, and all *p* values in the first three columns were equal to 0.001. Single asterisks indicate statistically significant differences (*p* < 0.05), whereas double asterisks indicate highly significant differences (*p* < 0.01).

## 4 Discussion

By focusing on the ventral outline of the snout and mouth, and on the relative size and position of the nostrils, this study captures key aspects of head morphology across nearly all extant shark species. This two‐dimensional approach, though simplified, enables an exceptionally broad taxonomic comparison in which even a restricted set of descriptors reveals consistent morphological patterns. Geometric morphometric analysis of both RO and RNO datasets revealed a clear taxonomic grouping. This pattern is consistent with the phylogenetic signal (*K* = 0.7621) calculated for the larger RO dataset, and with the r‐PLS analyses between modules, calculated for the RNO dataset, reinforcing the conclusion that head shape variation in sharks is largely constrained by evolutionary history, with ecological and behavioral factors playing a secondary role.

The morphospace obtained from the RO dataset (Figure 2) can be qualitatively compared with previously published morphospaces focusing on other cranial modules, such as the mandible [13] and the neurocranium [14]. Although these studies analyze different anatomical structures and are therefore not directly comparable in a quantitative sense, some recurring distributional patterns emerge. In López‐Romero et al. [13], the first two PCs account for approximately 57% of mandible shape variation, whereas in the present study, PC1 and PC2 together explain about 87% of the combined variation in snout and mouth rim shape. In both studies, the morphospace shows a partial subdivision between Squalomorphii and Galeomorphii, albeit with considerable overlap. In contrast to López‐Romero et al. [13], where Orectolobiformes and Squatiniformes do not overlap in mandible morphospace, the RO dataset shows an overlap between the phylogenetically distant Squatiniformes and the family Orectolobidae. Interestingly, both are absent from the RNO dataset because their nostrils are not visible in ventral view, which represents yet another convergent feature between them. This pattern suggests that similarity between these groups emerges specifically in the RO module, while remaining less evident in mandibular morphology, highlighting potential modular differentiation in cranial evolution. The overlap between Squatiniformes and Orectolobidae in the RO morphospace is congruent with the convergence signal detected by the *Ct* measures. Although proximity in morphospace does not automatically indicate convergent evolution, the *Ct* results suggest that these taxa have undergone directional evolutionary change toward similar RO configurations. Although previous work on fin morphology reported limited evidence of convergence between Orectolobiformes and Squatiniformes [40], our analyses of rostral traits recover a moderate but statistically supported signal.

The phylomorphospace presented by Gayford et al. [14] retrieves 57% of neurocranial shape variation along PC1 and PC2. Some distributional patterns are consistent across studies. For example, Sphyrnidae are separated from other Carcharhiniformes along PC2, and Pristiophoriformes are clearly isolated along PC1. The species occupying the extremes of the morphoclines along PC1 and PC2 partially coincide between datasets. In the present analysis, *Pliotrema warreni* and *Squatina nebulosa* represent the extremes of PC1, whereas in Gayford et al. [14], the extremes include *P. warrani* and the orectolobid *Sutorectus tentaculatus*. Given that Orectolobiformes and Squatiniformes, as mentioned, occupy overlapping regions of the morphospace in our analysis, this pattern further supports the idea that RO morphology reflects broader cranial diversification trends. Regarding PC2, *Eusphyra blochii* occupies one extreme in both studies. The opposite extreme differs between datasets, reflecting taxon sampling differences (e.g., *Chlamydoselacus anguineus* in the present study vs. the batoid *Pristis clavata* in Gayford et al. [14]).

Overall, these correspondences indicate that variation in the RO complex is integrated with overall cranial morphology, and that patterns of cranial diversification at the neurocranial and mandibular levels are at least partially mirrored in external rostral and oral shape.

Reconstruction of ancestral shapes in the present study is based exclusively on extant taxa; thus, the inferred ancestral shapes are necessarily conditioned on the present‐day morphospace and may not capture morphologies that were present in extinct lineages. The lack of fossil constraints is particularly relevant in sharks, whose fossil record is dominated by isolated teeth and is affected by taphonomic and depositional biases [21]. Moreover, the BM model assumes gradual, unbiased shape change with variance proportional to branch length. This assumption may be violated if rates of morphological evolution varied substantially across lineages or were decoupled from molecular rates, as has been suggested for sharks [21, 41]. Under such scenarios, our ancestral estimates are uncertain. Nevertheless, according to Compagno [42], as recalled by Bell [5], the ancestor of extant elasmobranchs likely possessed a short rostrum. The ancestral reconstructions obtained here, although restricted to Selachii and excluding Batoidea and with the caveat already presented, align with this earlier hypothesis by consistently indicating a compact rostral morphology. Moreover, several taxa interpreted as basal galeomorphs, such as *Paraorthacodus* sp. (BSPG 1996 I 31, Tithonian of Eichstätt, southern Germany) and *Synechodus* sp. (BSPG 1878 VI 6, Tithonian of Solnhofen, southern Germany) [43], show snout morphologies remarkably similar to our reconstructed ancestors of Selachii and Galeomorphii. Likewise, *Protospinax annectans* Woodward, 1918 (PBP SOL 8007, lower Tithonian of Solnhofen), interpreted as either a basal squalomorph or a basal lineage within Squatiniformes + Pristiophoriformes [44], strongly resembles the reconstructed Squalomorphii ancestor. These external comparisons, while not part of the quantitative analysis, provide an independent line of evidence supporting the plausibility of our ancestral reconstructions that should in any case be interpreted not as literal representations of historical shark morphologies, but as model‐based expectations that facilitate visualization of broad evolutionary trends in rostral shape across the shark phylogeny.

Several alternative modular hypotheses were tested on the RNO dataset to explore patterns of covariation among the analyzed structures. Across all analyses, partial least‐squares correlations revealed a strong and highly significant integration between snout, mouth, and nostril shape in Selachii, as well as within Galeomorphii and Squalomorphii. Incorporating phylogeny consistently reduced r‐PLS values, indicating that part of this integration reflects shared evolutionary history. However, strong and significant correlations persisted after phylogenetic correction, suggesting that integration is not solely driven by common ancestry, but likely reflects functional and structural coupling among these regions. The results consistently indicate differences in the strength of covariation between the mouth and the nostrils, with modules grouping the nostrils with the snout generally showing stronger integration than those in which the mouth was considered jointly with the nostrils. This pattern suggests that, within the rostral region, nostril shape tends to covary more tightly with overall rostral morphology, whereas the mouth appears comparatively more independent in its shape variation. No consistent differences in integration strength were found between Galeomorphii and Squalomorphii across all configurations. However, when significant differences emerged after phylogenetic correction, Galeomorphii consistently exhibited higher integration values (Table 4), suggesting a relatively more modular organization in Squalomorphii, which may be associated with their broader occupation of morphospace.

The correlations between the RNO region and ecological or functional variables yielded some interesting results. The shape of both the RO and RNO datasets did not correlate with swim type nor with relative swim speed. The overall three‐dimensional shape of the head may potentially influence swimming mode and performance [10]. However, the outline of the rostrum, as well as the position and width of the nostrils and mouth, does not appear to be correlated with these factors. Although the relative position of the nostrils with respect to the rostrum and mouth has been linked in the literature to a more sedentary or more active lifestyle [5], our data do not capture this functional association.

Morphotype also shows no significant association with RO and RNO shape variation, further suggesting that these structures are primarily influenced by local functional constraints rather than by overall body morphology or swimming mode. The need to accommodate a mouth adapted to the species’ feeding ecology [13] and sensory organs suited to its lifestyle may play a more important role in shaping head morphology than overall body form or locomotor features.

Allometric effects, measured as differences in shape relative to maxTL, explained a significant but very small fraction of variance (1%–3%). The same applies to the ecotype: The benthic, benthopelagic, and pelagic habits explained 3%–4% of the variance.

Among the ecological and functional variables tested, N‐lam of the olfactory organ showed the strongest association with ventral head shape, explaining approximately 20% of the variance, although this variable is currently available for only 53 species. This result suggests a potential link between external rostral morphology and the internal architecture of the olfactory system. Species of Sphyrnidae, characterized by extremely expanded snouts and unusually high lamellar counts [4], strongly influence this relationship. When hammerhead sharks are excluded from the analysis, the explained variance decreases to approximately 8% and loses statistical significance, indicating that the overall signal is driven in large part by these morphologically extreme taxa.

Nevertheless, the association remains biologically plausible. An increased number of lamellae implies greater structural complexity within the olfactory chamber and may require specific spatial arrangements to optimize water flow across the sensory epithelium. Because water intake and flow dynamics depend on the geometry of the nostrils, their spacing relative to the mouth, and the ventral contour of the rostrum, variation in N‐lam could be expected to covary with external RNO morphology [37, 38, 39]. Experimental and anatomical studies in sharks and other fishes have emphasized the importance of lamellae in directing intranasal water flow [37, 38, 39, 45], supporting the idea that hydrodynamic constraints may link internal sensory architecture with external rostral shape.

Although the current dataset is limited, these results are in agreement with the correlation between the rostrum and the nasal capsules found by Gayford et al. [14], and they raise the possibility that olfactory system complexity has played a role in shaping ventral head morphology in at least some shark lineages. Expanding comparative data on lamellar counts and olfactory chamber morphology across a broader taxonomic sample of Selachii would allow a more rigorous evaluation of this hypothesis and may reveal whether the pattern observed here reflects a general evolutionary trend or is primarily driven by extreme clades such as Sphyrnidae.

Likewise, refining ecological parameters may reveal additional patterns and correlations that remain undetected in the present analysis.

Overall, the analyses presented here show that the morphology of the snout and ventral facial openings in sharks is highly conserved across most of Selachii, and very similar to the hypothesized ancestral shape. Some extreme lineages show convergent morphologies associated with their benthic lifestyle. Phylogeny emerges as the dominant factor shaping the RNO complex, stronger than size, habitat, or swimming mode. From the earliest stages of their evolutionary history, the basic morphology of the shark snout appears to have been highly suitable to their ecological role, and it has remained remarkably stable over time.

## Author Contributions

Stefano Aicardi: conceptualization (equal), data curation (equal), formal analysis (equal), investigation (equal). Giacomo Rosa: formal analysis (equal), investigation (supporting), writing – original draft preparation (supporting). Alessio Longo: investigation (supporting), writing – review and editing (equal). Matteo Bozzo: investigation (supporting), writing – review and editing (equal). Beatrice Risso: writing – review and editing (equal). Elisa Damonte: writing – review and editing (equal). Andrea Amaroli: writing – review and editing (equal). Simona Candiani: writing – review and editing (equal). Sara Ferrando: conceptualization (equal), data curation (equal), formal analysis (equal), funding acquisition (lead), visualization (lead), writing – original draft preparation (lead).

## Conflicts of Interest

The authors declare no conflicts of interest.

## Supporting information




**Figure S1**: nyas70323‐sup‐0001‐FigureS1.pdf

**Figure S2**: nyas70323‐sup‐0001‐FigureS2.png

**Figure S3**: nyas70323‐sup‐0001‐FigureS3.png

**Figure S4**: nyas70323‐sup‐0001‐FigureS4.png

**Figure S5**: nyas70323‐sup‐0001‐FigureS5.png

**Figure S6**: nyas70323‐sup‐0001‐FigureS6.png

**Supporting Table**: nyas70323‐sup‐0007‐TableS1.xlsx

**Supporting materials**: nyas70323‐sup‐0008‐FileS1.txt

## Data Availability

The data underlying this article will be shared on reasonable request to the corresponding author.

## Footnotes

1
http://researcharchive.calacademy.org/research/ichthyology/catalog/fishcatmain.asp
