# Supplementary figures and images for: Evolutionary Stability of the Shark Snout: Geometric Morphometrics of Ventral Facial Openings
Source: Ann N Y Acad Sci. 2026 Jun 16;1560(1):e70323. doi: 10.1111/nyas.70323 (PMC13271047; doi:10.1111/nyas.70323)

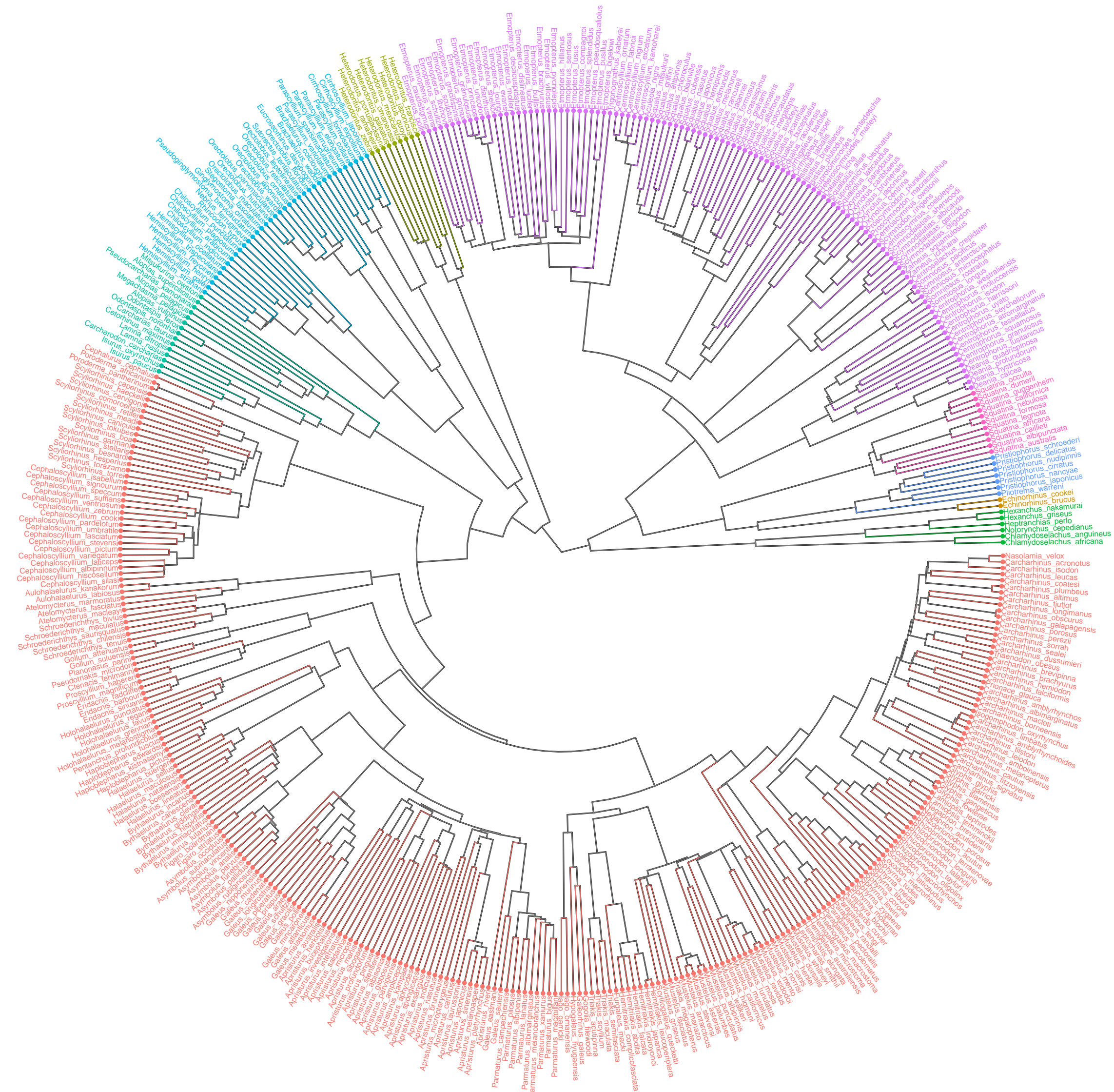

Supplement: Supplementary file 1 — Figure S1: nyas70323‐sup‐0001‐FigureS1.pdf [file NYAS-1560-0-s007.pdf]

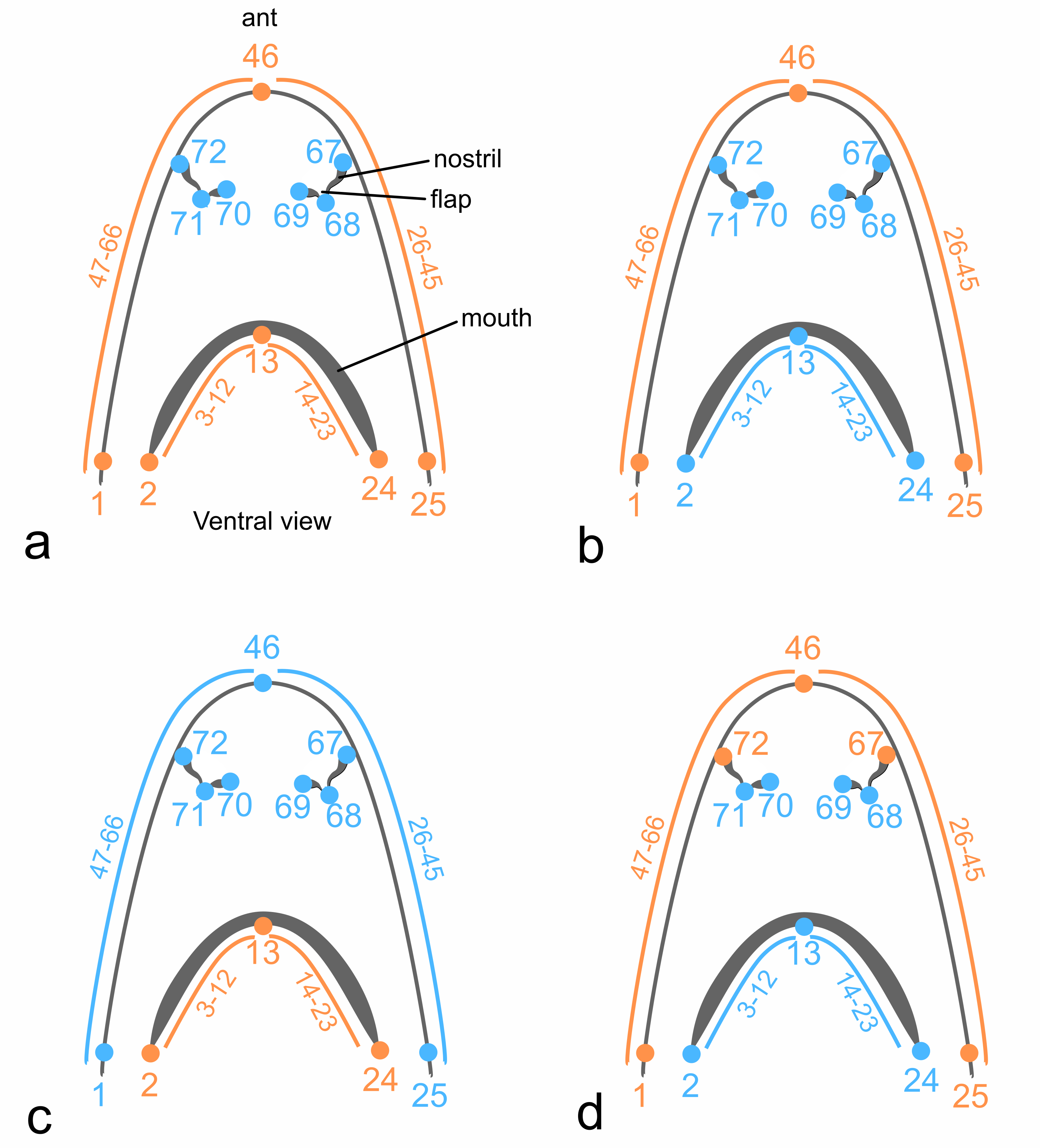

Supplement: Supplementary file 2 — Figure S2: nyas70323‐sup‐0001‐FigureS2.png [file NYAS-1560-0-s004.png]

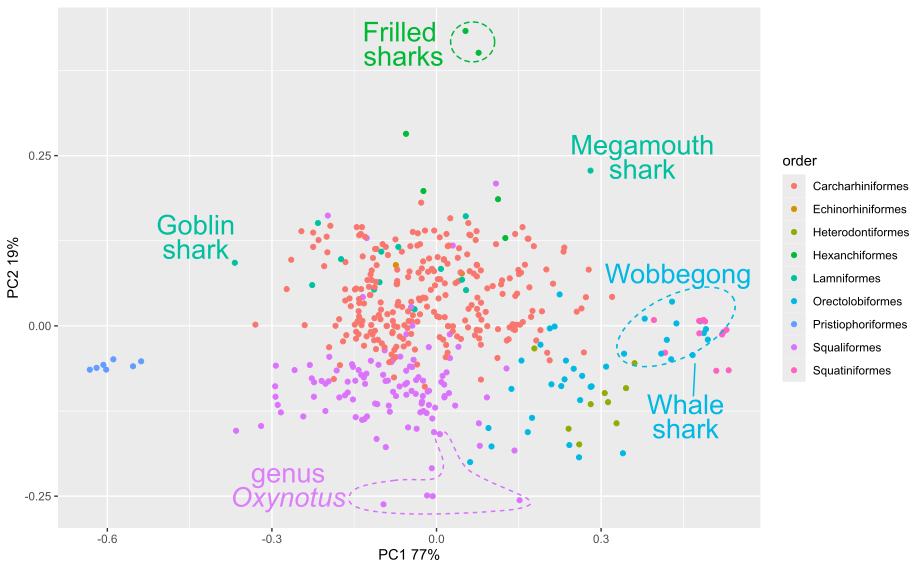

Supplement: Supplementary file 3 — Figure S3: nyas70323‐sup‐0001‐FigureS3.png [file NYAS-1560-0-s008.png]

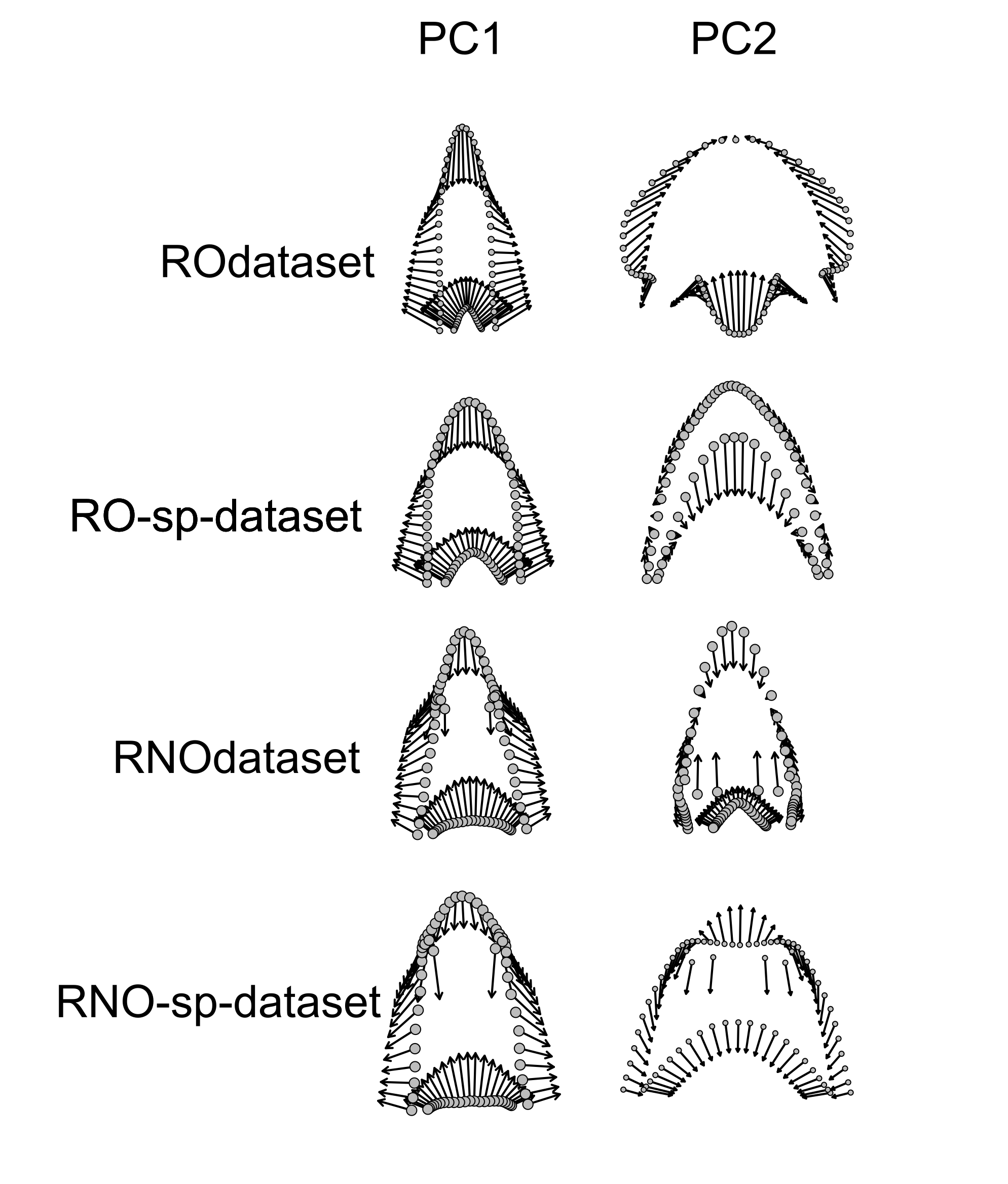

Supplement: Supplementary file 4 — Figure S4: nyas70323‐sup‐0001‐FigureS4.png [file NYAS-1560-0-s006.png]

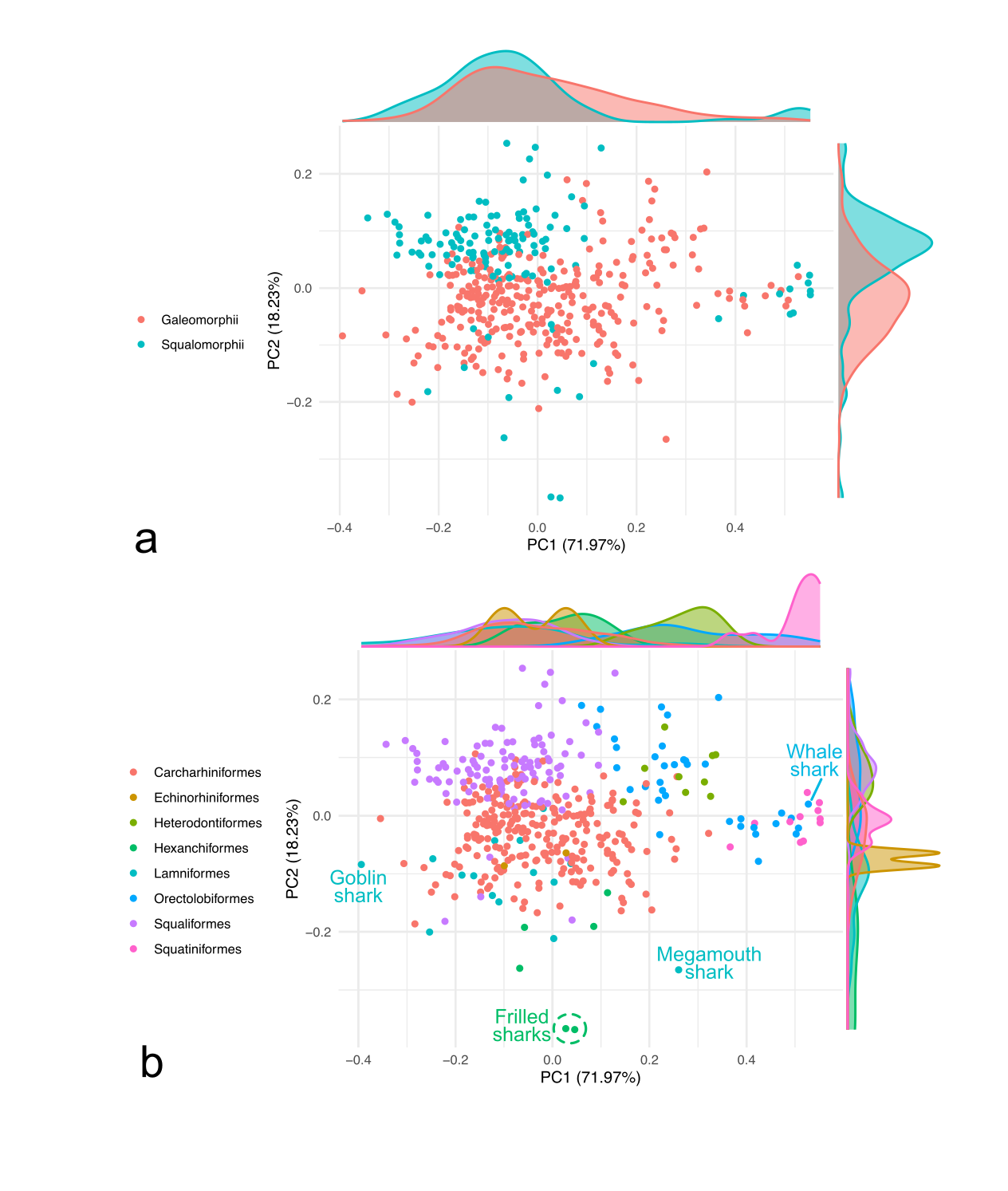

Supplement: Supplementary file 5 — Figure S5: nyas70323‐sup‐0001‐FigureS5.png [file NYAS-1560-0-s003.png]

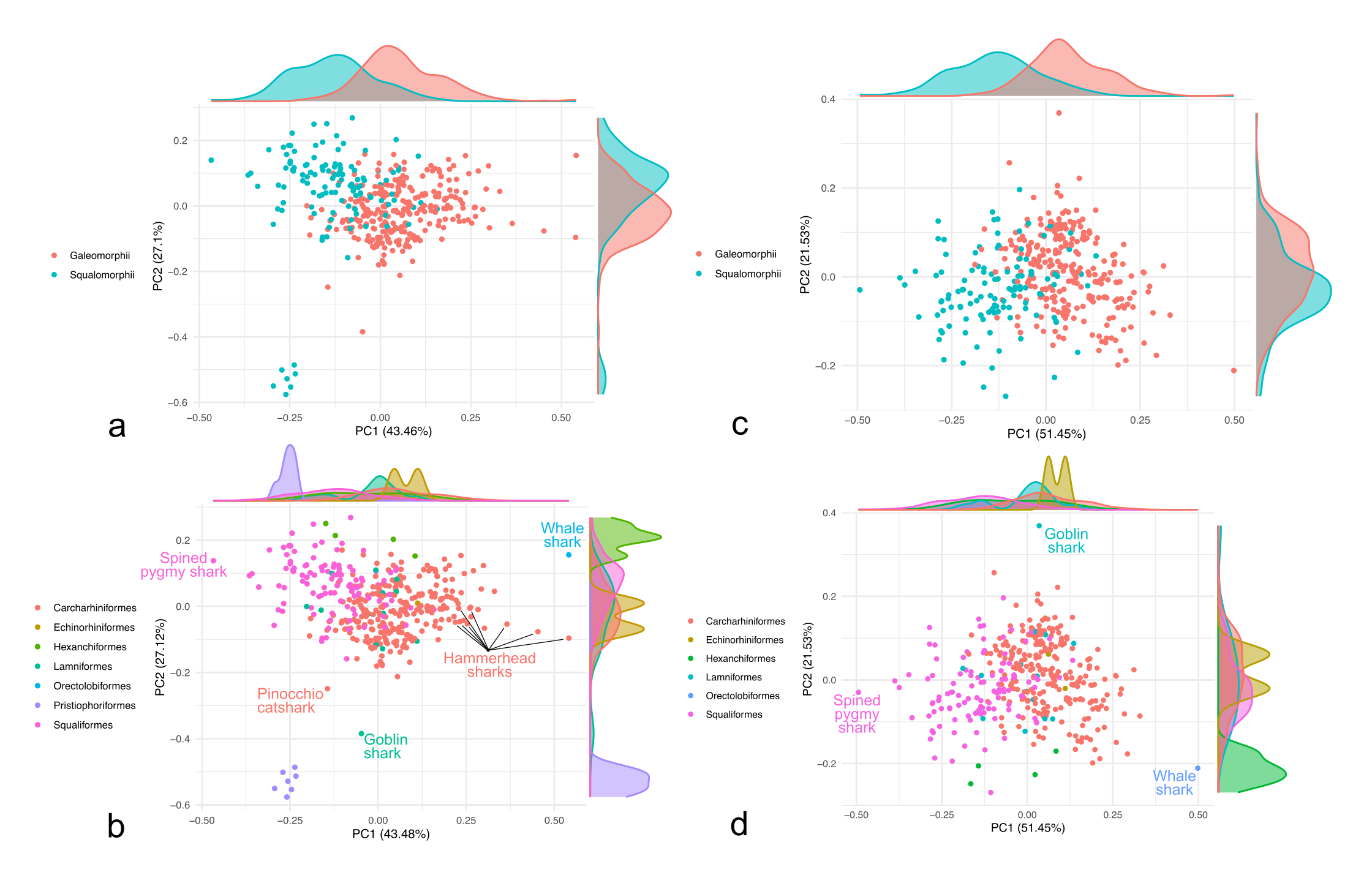

Supplement: Supplementary file 6 — Figure S6: nyas70323‐sup‐0001‐FigureS6.png [file NYAS-1560-0-s001.png]
